# miR-424-5p shuttled by bone marrow stem cells-derived exosomes attenuates osteogenesis via regulating WIF1-mediated Wnt/β-catenin axis

**DOI:** 10.18632/aging.203169

**Published:** 2021-07-06

**Authors:** Yongkun Wei, Huiling Ma, Haiqing Zhou, Hanrong Yin, Jie Yang, Yongcai Song, Binhui Yang

**Affiliations:** 1 Departments of Orthopedics and Pathology, 3201 Hospital, Hanzhong 723000, Shaanxi, China; 2 Hanzhong Vocational and Technical College, Hanzhong 723002, Shaanxi, China

**Keywords:** bone marrow stem cells, exosomes, osteogenesis, Wnt/β-catenin

## Abstract

Emerging evidence proves that exosomes contain specific microRNAs(miRNAs) contribute to osteogenic differentiation of bone marrow stem cells (BMSCs). However, the role and mechanism of bone marrow stem cells (BMSCs)-derived exosomes overexpressing miR-424-5p in osteoblasts remains unclear. Firstly, the BMSCs-derived exosomes were isolated, and identified by Western blot with the exosome surface markers CD9, CD81 and CD63. Quantitative real-time polymerase chain reaction (qRT-PCR) was applied to detect the level of miR-424-5p in exosomes, and western blot was implemented to verify the WIF1/Wnt/β-catenin expression. The binding association between miR-424-5p and WIF1 was determined by the dual-luciferase reporter gene assay. Functional enhancement experiments were adopted to determine the role of exosome-carried miR-424-5p and WIF1/Wnt/β-catenin in osteogenic differentiation. ALP staining was adopted, and levels of RUNX2, OCN, and OPN were monitored using qRT-PCR to determine osteogenic differentiation. As a result, *In vivo* experiments showed that RUNX2, OCN and OPN levels decreased and the ALP activity was dampened after miR-424-5p overexpression in exosomes. Besides, exosomes overexpressing miR-424-5p attenuated osteogenic development via WIF1/Wnt/β-catenin. Our findings may bring evidence for miR-424-5p as a new biomarker for the treatment of osteoporosis.

## INTRODUCTION

Osteoporosis is a disease characterized by bone loss, microstructural degeneration, and brittle fractures [1]. Bone marrow stem cells (BMSCs) could differentiate into diversified cell types, such as osteogenesis, chondrogenesis and lipogenesis lineages. BMSCs osteogenesis can be induced by paracrine cytokines, thus promoting bone formation and regeneration [2]. BMSCs are key components of new bone formation, and recent studies have shown that BMSCs-derived exosomes containing miRNAs mediate the development of osteogenesis. Therefore, it is critical to study the effect of miRNAs shuttled by exosomes on osteogenesis.

Exosomes are endocytic vesicles with a length of 30-120 nm, which are involved in intercellular communication and transmission of the protein and RNA. Vast studies have demonstrated that molecular components in exosomes, especially proteins and miRNAs [3]. The latest studies have manifested that miRNAs in exosomes regulate the skeletogeny, dampen osteoclast activity and improve fracture repair, which may be a promising treatment target for osteoporosis [4, 5]. Jiang LB et al. found that miR-21 expression in MSCs exosomes, extracted from osteoporosis patients, is significantly higher than that from healthy adults. Meanwhile, miR-21 could down regulate SMAD7, which resulting in inhibiting osteogenic differentiation [6]. Besides, exosomes overexpressing miR-122-5p down-regulate SPRY2 via the RTK/Ras/mitogen-activated protein kinase (MAPK) pathways, thereby alleviating osteonecrosis of the femoral head (ONFH) [7]. As one of core miRNAs for tension-induced bone formation, studies have proved that miR-424-5p is related to the pathology of subchondral bone (SCB) sclerosis. But whether miR-424-5p overexpressed BMSCs-derived exosomes enhance the therapeutic effect of bone regeneration remain unclear. Based on the above studies, we wondered whether BMSCs-derived exosomes could be used as the vector of osteogenic miRNAs, especially the miR-424-5p.

Wnt inhibitor (WIF)-1 is a member of the Wnt protein secretion regulator, and it directly interacts with various Wnt ligands and attenuates their combination with membrane-bound receptors [8]. The Wnt/β-catenin pathway, also known as the classical Wnt pathway, is one essential pathway during osteogenesis [9]. Studies have proved that the WIF1/Wnt/β-catenin pathway contributes to osteogenic differentiation. Additionally, when sclerosin antibody (Scl-Ab) is used to elevate bone formation and bone mass, repeated drug administration is found to inhibit bone formation, which is associated with enhanced Wnt antagonist expression [10]. Besides, An JH et al. found WIF-1 impairs ALP activity and changes in mitochondrial biogenesis in Wnt-3A conditional drug (CM)-induced osteogenic differentiation [11]. On the other hand, miRNAs are confirmed to be involved in disease progression by regulating the WIF1/Wnt/β-catenin pathway. For instance, miR-590-3p promotes the progression of colon cancer by down-regulating WIF1 to activate Wnt/β-catenin [12]. According to the above studies, we could conducted that WIF1/Wnt/β-catenin pathway plays a vital role in osteogenic differentiation, and BMSCs-derived exosomes overexpressing miRNAs also regulating bone regeneration. Based on these, the manuscript hypothesis that BMSCs-derived exosomes overexpressing miR-424-5p enhance bone regeneration through the WIF1/Wnt/β-catenin pathway remains elusive.

Here, we intended to investigate the function of BMSCs-derived exosomes in osteoblast differentiation and the role of miR-424-5p in this process. We extracted exosomes from BMSC cell lines, and discovered that BMSCs-derived exosomes inhibited osteoblast differentiation by up-regulating miR-424-5p, which resulting in suppressing WIF1/Wnt /β-catenin.

## MATERIALS AND METHODS

### Specimen collection and cell culture

The experiment of this study was authorized by the Research Ethics Committee of the Departments of Orthopedics and Pathology, 3201 Hospital. All patients who provided specimens for this study signed informed consent. Bone marrow specimens were obtained from 10 femoral head tissues with osteoporosis that were discarded during total hip arthroplasty (THA). Human BMSCs were isolated by the whole bone marrow adherent method. Then, the cells were cultured in RPMI1640 (Thermo Fisher Scientific, MA, USA) supplemented with 10% fetal bovine serum (FBS) (Thermo Fisher Scientific, MA, USA) and 1% penicillin/streptomycin (Invitrogen, CA, USA) at 37° C with 5% CO_2_. During the logarithmic growth phase, 0.25% trypsin (Thermo Fisher HyClone, Utah, USA) was used for trypsinization and passage. Subsequently, BMSCs were inoculated into 100-mm tissue culture dishes and cultured up to 80% fusion rate to prepare exosomes. Afterward, the cells were cultured in a medium containing 2% exosomes-free serum (obtained through centrifugation at 4° C for 14 hours at 100000 rpm of serum) for 2 days.

### Harvesting and identification of BMSCs-derived exosomes

As previously reported [13], the cell supernatant was collected and centrifuged at 4° C at 300 rpm (10 min), 1200 rpm (20 min), and 10,000 rpm (30 min), respectively. Then, the final supernatant was centrifuged at 4° C at 100,000 rpm for 1 hour. After the supernatant was removed, the exosome sediments were washed with plenty of cold PBS and centrifuged at 100,000 rpm at 4° C for 1 hour. Subsequently, the residual liquid was decanted and sucked out to obtain exosomes, which were reserved at -80° C until use. Finally, exosomes were examined with transmission electron microscopy (TEM, Tecnai, FEI, Hillsboro, OR, USA), and western blot was used to monitor exosome surface markers CD9 (1: 1000, Abcam, Cambridge, UK), CD63 (1: 1000, Abcam) and CD81 (1: 1000, Abcam).

### Osteogenic differentiation *in vitro*

For osteoblast differentiation, BMSCs were inoculated in 12-well plates at 5×10^4^ cells/well for 24 hours before induction. Then, osteogenic differentiation was induced by culturing BMSCs in osteogenic differentiation medium (ODM, containing 20 mM β-glycerophosphate, 50 μg/mL ascorbic acid and 100 nM dexamethasone). The medium was altered every 3-4 days. Alizarin red staining (ARS) and qRT-PCR were conducted to determine the impact of exosomes on osteogenic differentiation. In addition, 2 μg/mL BMSCs-derived exosomes were added to BMSCs in the ODM to determine its effect on differentiation.

### Cell transfection

Human BMSCs at the logarithmic growth stage were seeded into 6-well plates (5×10^5^/well). When the fusion rate of the cells reached 50%-60%, pcDNA empty vector (NC), pcDNA-WIF1 (WIF1), miR-424-5p inhibitor (miR-424-5p inhibitor group) or NC(NC group) synthetized by GenePharma (Shanghai, China) were transfected into BMSCs following the specification of the FuGENE®HD Transfection Reagent (Roche, Shanghai, China). After being incubated for 48 hours, the cells were collected, and the RNA or protein expression was tested by quantitative real-time polymerase chain reaction (qRT-PCR) and western blot.

### Alizarin Red S (ARS) staining and quantitative analysis

ARS staining was applied to examine bone formation of BMSCs. In short, BMSCs were incubated with ARS staining buffer (Cyagen Bioscience, USA) for 20-30 min. Then, they were examined under an optical microscope (Nikon, Tokyo, Japan) to determine their salinity. Upon ARS dissolution, BMSCs were incubated with 100 mM cetylpyridinium chloride (Sigma, St Louis, MO, USA) for 1 hour. Finally, the optical density of the released ARS was measured at 570 nm with a microplate reader (Tecan, Mannedorf, Zurich, Switzerland).

### Alkaline phosphatase (ALP) staining

The BMSCs were cultured in 24-well plates, and then the medium was discarded. Afterward, the cells were washed with phosphate-buffered saline (PBS), fixed with 95% ethanol, stained with ALP solution and cultured for 4 hours in an incubator at 37° C. Then, 2% cobalt sulfide and ammonium sulfide (Tianjin Tianli Chemical Reagent Co., Ltd, Tianjin, China) were added. Subsequently, the cells were incubated with 10 mM p-nitrophenyl phosphate (Meilunbio, Dalian, China) for 30 min for quantitative analysis. Finally, the release of p-nitrophenol was determined by the ALP colorimetric kit (BioVision, USA) to evaluate the ALP activity. The absorbance was measured at 420 nm with a microplate reader (Tecan, Mannedorf, Zurich, Switzerland).

### QRT-PCR

Total cellular RNA was extracted with the TRIzol reagent, and then it was reverse-transcribed into cDNA with PrimeScript™ RT Reagent kit (Invitrogen, Shanghai, China) as the manufacturer's specifications. Bio-Rad CFX96 quantitative PCR system and SYBR were used for qRT-PCR following the manufacturer's regulations. The conditions for PCR were as follows: Initial denaturation for 5 min at 95° C, denaturation for 15 s at 95° C, and annealing for 30 s at 60° C. GAPDH and U6 served as internal references of genes and miRNAs, respectively. The 2 ^(-ΔΔCt)^ method was adopted for statistics. Each experiment was done in triplicate. The specific primer sequences were in Table 1.

**Table 1:** The specific primer sequences were as follows.

**Target genes**	**Forward (5 '-3')**	**Reverse (5 '-3')**
miR-424-5p	AACCACTCAGCAGCAATTCATGT	CAGTGCAGGGTCCGAGGT
RUNX2	AGATGATGACACTGCCACCTCTG	GGGATGAAATGCTTGGGAACTGC
OPN	CTCCATTGACTCGAACGACTC	CAGGTCTGCGAAACTTCTTAGAT
OCN	TGAGAGCCCTCACACTCCTC	CGCCTGGGTCTCTTCACTAC
ALP	TTGTGCCAGAGAAAGAGAGAGAGA	GTTTCAGGGCATTTTTCAAGGT
U6	CGCTTCGGCAGCACATATAC	TTCACGAATTTGCGTGTCAT
GAPDH	ATCCCATCACCATCTTCC	GAGTCCTTCCACGATACCA

### Western blot and co-immunoprecipitation

Total protein was obtained from human BMSCs with RIPA lysis buffer (Beyotime Biotechnology, Shanghai, China). Protein quantification was carried out with the Bradford method, and the protein samples were boiled for 5 min, cooled on ice and centrifuged for 30 s. Then, the supernatant was taken for polyacrylamide gel electrophoresis, and transferred to the polyvinylidene fluoride (PVDF) membranes at 100 V for 1 hour. Afterward, the membranes were blocked with 5% skimmed milk at room temperature (RT) for 1 hour, and then incubated with the primary anti-ALP (ab83259, 1:1000, Abcam, MA, USA), RUNX2 (ab76956, 1:1000), OPN (ab214050, 1:1000, Abcam), OCN (ab93876, 1:500, Abcam), WIF1 (1:1000, ab155101, Abcam), Wnt (1:1000, ab219412, Abcam), β-catenin (1:1000, ab32572, Abcam) or β-actin (ab8226, 1:1000) overnight at 4° C. Next, the membranes were rinsed with TBST twice, and incubated with the luciferin-labeled secondary antibody for 1 hour at RT. After washing the membrane for three times, ECL chromo-developing agent (Millipore, Billerica, MA, USA) was applied for exposure, and the membrane scanner was adopted for imaging.

For co-immunoprecipitation, the cells were rinsed with PBS and lysed for 20 min in a 4° C lysis buffer containing 50 mM Tris-HCl, 150 mM NaCl, 1% NP-40, 10% glycerol, 0.1% SDS, and 2 mM Na_2_EDTA. The lysates were then removed through centrifugation, and the protein was immunoprecipitated with the affinity antibodies and A+G agarose beads (Beyotime, China) at 4° C. The immunoprecipitation was washed 3 times with 1 mL lysis buffer, and then analyzed by standard western blot.

### Dual-luciferase reporter assay

The TargetScan software predicted that WNT3a was a potential target of miR-424-5p. Wild type and mutant WNT3a-3’UTR reporter gene plasmids were constructed, and miR-424-5p mimics and their negative controls were transfected into BMSCs. The dual-luciferase reporter assay was caried out 48 hours later following the experimental specifications (Promega, Madison, WI, USA), and the relative fluorescence intensity of different treatment groups was determined according to the ratio of firefly fluorescence intensity/renilla fluorescence intensity examined by the microplate reader.

### RNA binding protein immunoprecipitation (RIP) assay

RIP was implemented with Magna RIP Kit (EMD MilliPore, Billerica, MA, United States) as per manufacturer's guidelines. After the cells were treated with RIP lysis buffer, human Anti-Ago -2 antibody (microporous) or the control antibody (normal mouse immunoglobulin, Micropores) was added and incubated overnight at 4° C. QRT-PCR was conducted to verify the WNT level.

### Statistical analysis

SPSS17.0 statistical software (SPSS Inc., Chicago, IL, USA) was employed for analysis. Measurement data were presented as mean ± standard deviation (x±s). One-way ANOVA was adopted for multi-factor comparison, and the *t* test was employed for pair-wise comparison. *P*<0.05 indicated statistical significance.

### Ethics statement

Our study was approved by the Ethics Review Board of 3201 Hospital.

### Data availability statement

The data sets used and analyzed during the current study are available from the corresponding author on reasonable request.

## RESULTS

### Identification and characterization of BMSCs-derived exosomes

**Figure 1:**
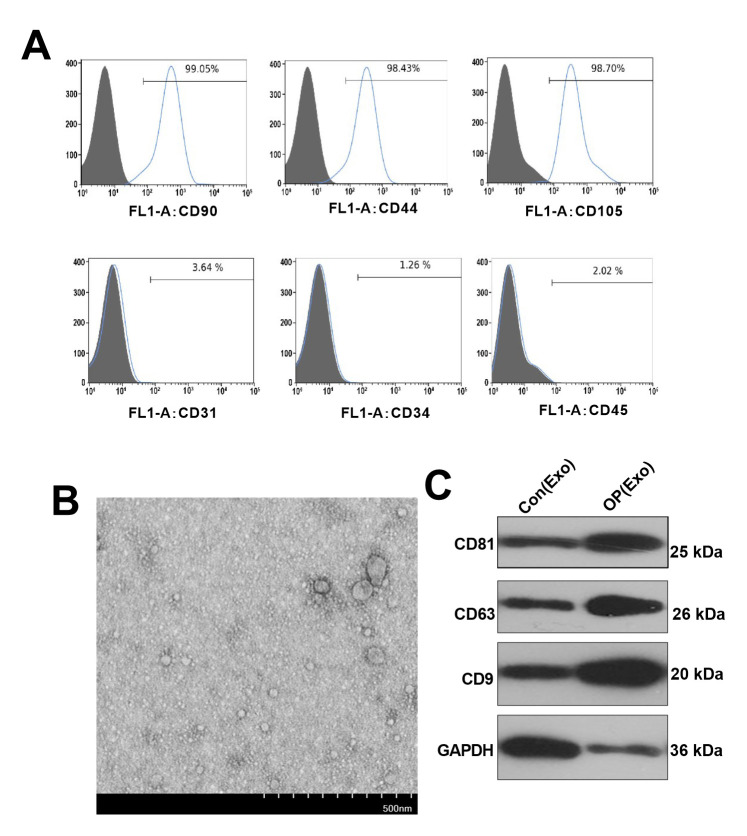
**Identification and characterization of BMSCs-derived exosomes.** (**A**) Flow cytometry was conducted to test the surface markers of BMSCs. (**B**) Exosomes were extracted from BMSCs by TEM identification. (**C**) Western blot was conducted to monitor the exosome surface markers.

Flow cytometry analysis with cell surface specific markers was used to identify BMSCs (Figure 1A). It was found that CD90, CD44 and CD105 were positive, while CD31, CD34 and CD45 were negative in BMSCs. BMSCs-derived exosomes were isolated through centrifugation (Figure 1B), and the purified exosomes were observed by TEM. Western blot confirmed that BMSCs-derived exosomes expressed and enriched with known exosome markers CD9, CD63 and CD81 (Figure 1C).

### BMSCs-derived exosomes inhibited osteogenesis

**Figure 2:**
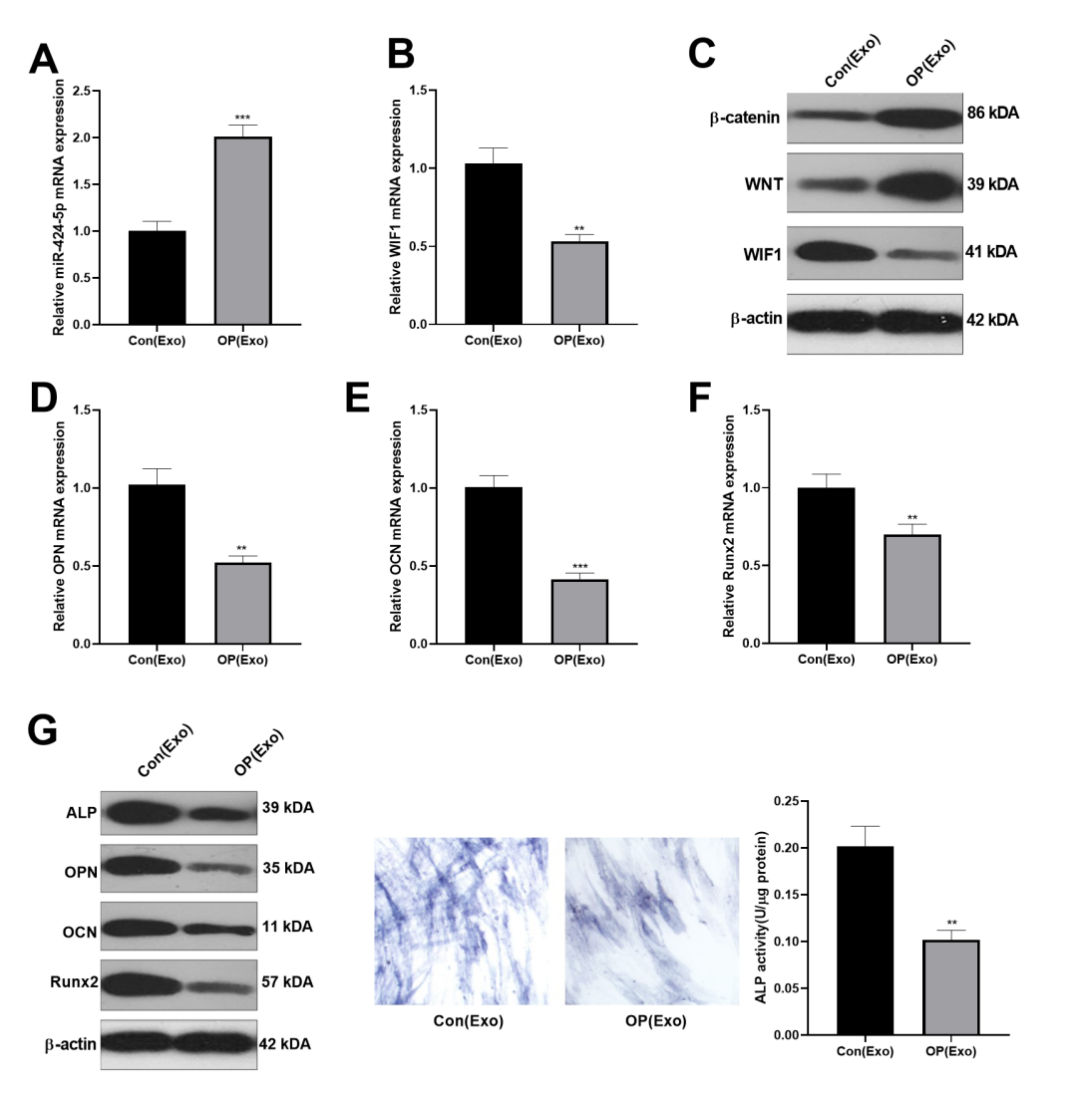
**Inhibitory effect of BMSCs-derived exosomes on osteogenesis.** Osteogenic BMSC cells were treated with exosomes from normal bone and osteoporosis patients for 7 days, respectively. (**A**, **B**) The miR-424-5p and WIF1 levels were verified by qRT-PCR. (**C**) Western blot was implemented to examine the protein expression of WIF1/Wnt/β-catenin. (**D**–**F**) The expression of OPN, OCN and Runx2 mRNAs were compared by qRT-PCR. (**G**) Western blot was implemented to examine the protein expression of OPN, OCN and Runx2. (**H**) ALP staining. ***P<0.01, ***P<0.001 (vs. Con(Exo)).

Osteogenic BMSC cells from patients were treated with normal BMSCs-derived exosomes for 7 days. The levels of miR-424-5p and WIF1 were testified by qRT-PCR, which confirmed that miR-424-5p was overexpressed, while WIF1 was knocked down in exosomes from osteoporosis patients (Figure 2A, 2B). Then, we adopted western blot to determine the WIF1/Wnt/β-catenin protein expression, and discovered that compared with that in exosomes extracted from normal cells, WIF1 was knocked down, while Wnt and β-catenin were overexpressed in exosomes from osteoporosis patients (Figure 2C). Subsequently, the mRNA expression of OPN, OCN, and Runx2 in BMSCs were verified by qRT-PCR, and the results indicated that their levels in exosomes extracted from osteoporosis patients were lower than those in the control group (Figure 2D–2F), which was further demonstrated by western blot (Figure 2G). Besides, ALP staining showed that compared with BMSCs treated with exosomes extracted from normal, those treated with exosomes from osteoporosis patients had less blue cytoplasmic granules and decreased ALP (Figure 2H), suggesting that exosomes derived from BMSCs in osteoporosis patients inhibited osteogenesis.

### Dampening the miR-424-5p in exosomes enhanced osteogenic differentiation of BMSCs

**Figure 3:**
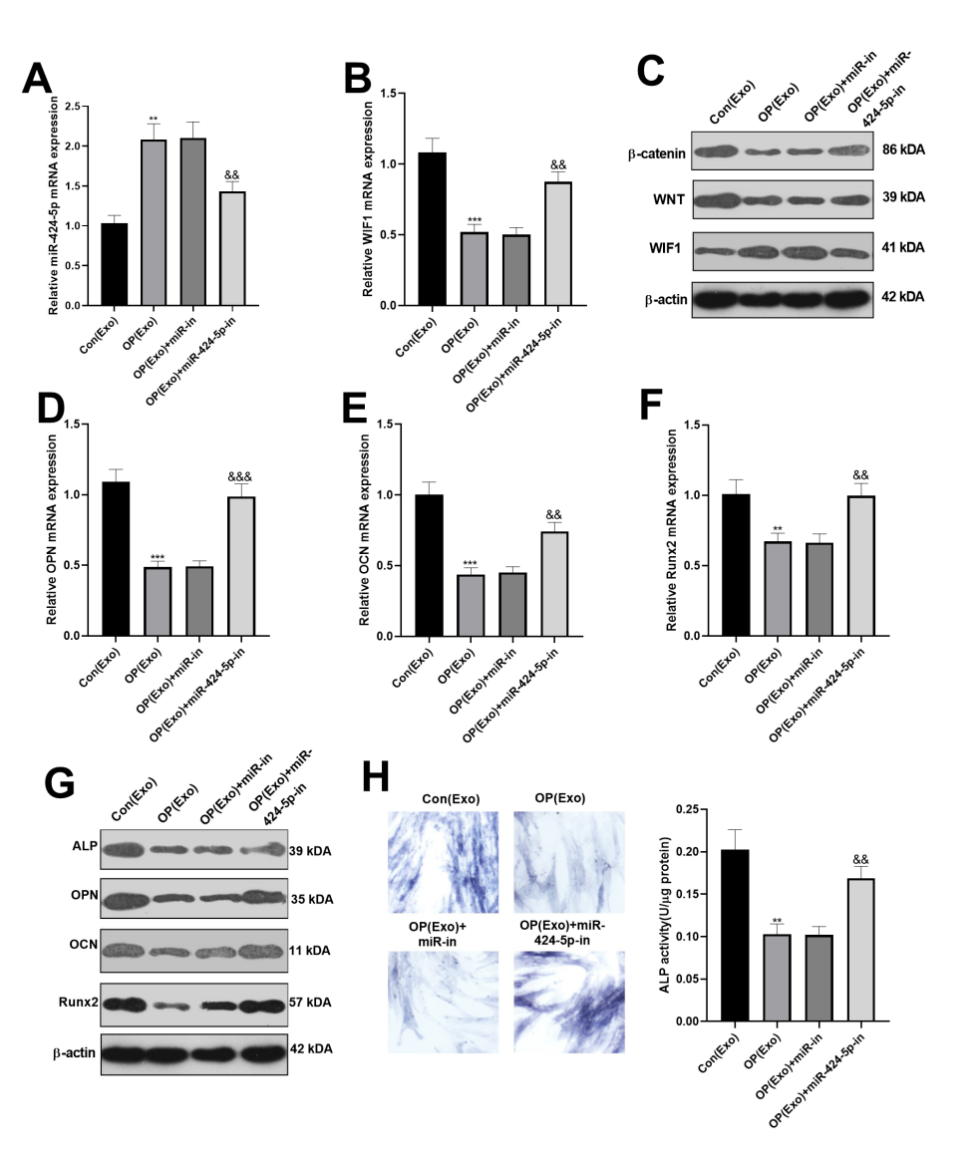
**Dampening miR-424-5p expression in exosomes enhanced osteogenic differentiation of BMSCs.** MiR-424-5p inhibitors were transfected into BMSCs-derived exosomes, which were then co-cultured with BMSCs for 7 days. (**A**, **B**) The expression of miR-424-5p and WIF1 were detected by qRT-PCR. (**C**) Western blot was adopted to test the protein expression of WIF1/Wnt/β-catenin. (**D**–**F**) QRT-PCR was implemented to verify the mRNA expression of OPN, OCN and Runx2. (**G**) Western blot was employed to monitor the protein expression of OPN, OCN and Runx2. (**H**) ALP staining. **P<0.01, ***P<0.001 (vs. Con(Exo)). &&*P*<0.01, &&&*P*<0.001 (vs. Con(Exo)+miR-in).

BMSCs-derived exosomes with miR-424-5p inhibitors were obtained, and then co-cultured with osteogenic BMSCs for 7 days. Then, the miR-424-5p and WIF1 expression were examined by qRT-PCR. As a result, compared with the OP(Exo)+ miR-NC group, miR-424-5p was knocked down and WIF1 was overexpressed after transfection with miR-424-5p inhibitors (Figure 3A, 3B). Meanwhile, western blot was performed to test the WIF1/Wnt/β-catenin expression, and it was found that compared with the OP(Exo)+ miR-NC group, WIF1 was overexpressed, while Wnt and β-catenin were knocked down after transfection of miR-424-5p inhibitors (Figure 3C). Subsequently, the mRNA expression of OPN, OCN, and Runx2 in BMSCs were compared by qRT-PCR, and the results illustrated that their levels were notably higher than those of the OP(Exo)+miR-NC group after transfection of miR-424-5p inhibitors (Figure 3D–3F). Also, western blot demonstrated the protein expression changes of OPN, OCN, and Runx2 were similar with that in mRNA (Figure 3G). Furthermore, ALP staining proved that compared with OP(Exo)+miR-NC group, the BMSCs transfected with miR-424-5p inhibitors had more blue cytoplasmic granules, accompanied by ALP elevation (Figure 3H), suggesting that dampening miR-424-5p expression in exosomes enhanced osteogenic differentiation of BMSCs.

### miR-424-5p targeted WIF1

**Figure 4:**
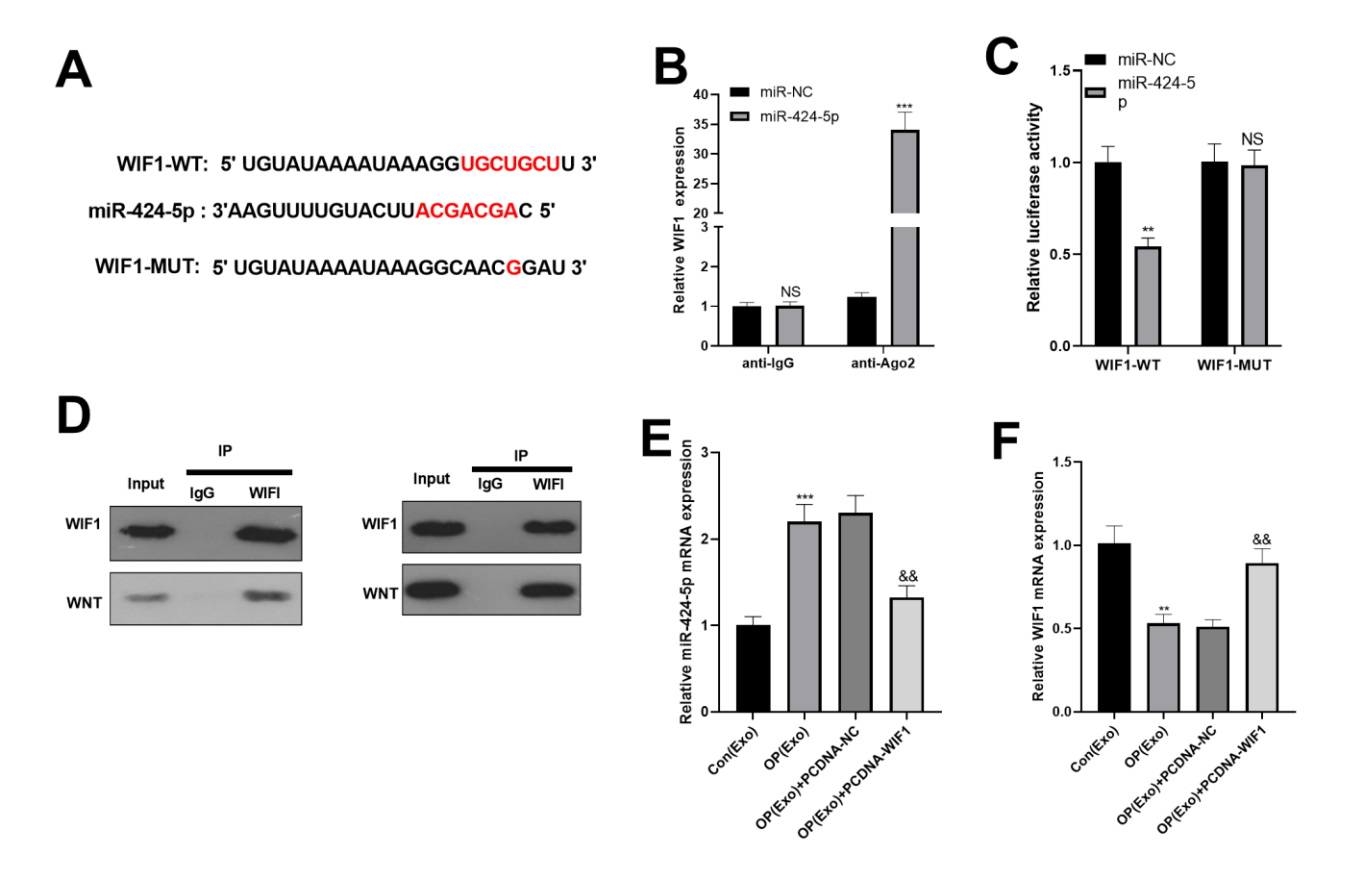
**miR-424-5p targeted WIF1.** (**A**) The downstream molecules of miR-424-5p were predicted by StarBase database (https://web.archive.org/web/20110222111721/http://starbase.sysu.edu.cn/), and miR-424-5p was found to be the target of WIF1. (**B**) The binding association between miR-424-5p and WIF1 was testified by dual-luciferase reporter assay. (**C**) RIP was conducted to determine the targeting correlation between miR-424-5p and WIF1. (**D**) IP was applied to examine the association between WIF1 and Wnt. (**E**, **F**) MiR-424-5p and WIF1 levels were tested by qRT-PCR. NS*P>*0.05, **P<0.01, ***P<0.001 (vs. miR-NC, vs. Con(Exo)). &&*P*<0.01, &&&*P*<0.001 (vs. Con(Exo)).

We adopted the online websites to search the target genes of miR-424-5p. Interestingly, StarBase (https://web.archive.org/web/20110222111721/http://starbase.sysu.edu.cn/) proved that WIF1 was a target of miR-424-5p (Figure 4A). In addition, dual-luciferase reporter assay proved that miR-424-5p bound to the wild-type WIF1 (Figure 4B). Hence, we conducted RIP to further clarify the relationship between WIF1 and miR-424-5p. As a result, after BMSCs were transfected with miR-424-5p, the amount of WIF1 precipitated in the Ago2 antibody group was far more than that in the IgG group, suggesting that WIF1 bound to Ago2 through miR-424-5p (Figure 4C). Subsequently, immunoprecipitation showed that WIF1 bound to WNT, and WIF1 was expressed in WNT, while not in IgG (Figure 4D). Finally, we transfected WIF1 overexpressed plasmids in BMSCs-derived exosomes. The result manifested that after transfection of WIF1 overexpressed plasmids, the miR-424-5p mRNA was down-regulated, while the WIF1 mRNA was up-regulated (Figure 4E, 4F). The above findings illustrated that miR-424-5p targeted WIF1 and was negatively correlated with it.

### BMSCs-derived exosomes attenuated osteogenesis via regulating miR-424-5p/WIF1

**Figure 5:**
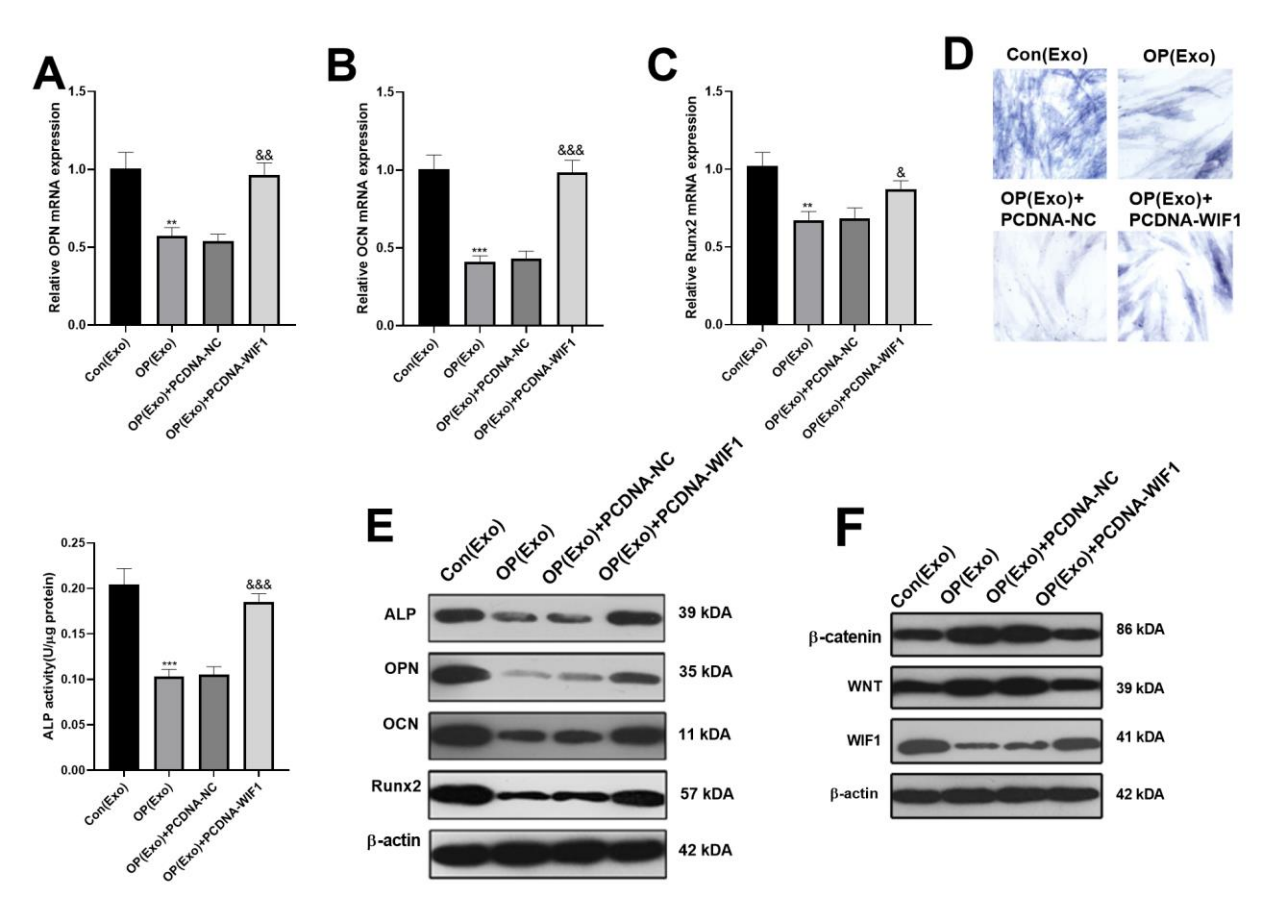
**BMSCs-derived exosomes inhibited osteogenesis via miR-424-5p/WIF1.** The WIF1 overexpressed plasmid was transfected into BMSCs-derived exosomes. (**A**–**C**) The expression of OPN, OCN and Runx2 were compared by qRT-PCR. (**D**) ALP staining was used to examine ALP activity. (**E**) Western blot was applied to determine the protein expression of OPN, OCN and Runx2. (**F**) The protein expression of WIF1/Wnt/β-catenin was verified by western blot. **P<0.01, ***P<0.001 (vs. Con(Exo)). &&P<0.01, &&&P<0.001 (vs. OP(Exo)+PCDNA-NC).

BMSCs were divided into 4 groups based on different treatment methods, namely Con(Exo), OP(Exo), OP(Exo)+PCDNA-NC and OP(Exo)+PCDNA-WIF1. Interestingly, the mRNA levels of OPN, OCN and Runx2 in the OP(Exo) group were distinctly lower than that in the Con(Exo) group. Beside, the mRNA expression of OPN, OCN and Runx2 in the OP(Exo)+PCDNA-WIF1 group was increased compared with the OP(Exo)+PCDNA-NC group. However, mRNA levels of these genes did not change significantly compared with the Con(Exo) group (Figure 5A–5C). Similar results were obtained by ALP activity detection (Figure 5D). Also, western blot was implemented to compare the protein expression of OPN, OCN and Runx2, and the results were consistent with the gene expression trend (Figure 5E). Furthermore, western blot was used to monitor the expression of WIF1/Wnt/β-catenin. Interestingly, compared with the Con(Exo) group, WIF1 was down-regulated, while Wnt and β-catenin were up-regulated in the OP(Exo) group, and results were reversed after transfection of WIF1 overexpressed plasmid (Figure 5F). The above results suggested that WIF1 overexpression exosomes partially reversed the osteogenic effect of BMSCs-derived exosomes in osteoporosis patients.

## DISCUSSION

Osteoporosis is a frequent age-related disorder characterized by low bone mass and deteriorating bone microstructure, resulting in increased bone vulnerability and fracture risk. Osteoporosis is usually associated with an imbalance between osteoblasts and osteoclasts [14]. On the other hand, exosomes are heterogeneous groups of cell-derived membrane structures that mediate cross-talk between cells. The latest research has shown a strong link between exosomes and bone homeostasis [15]. Proteins and miRNAs in bone cell-derived exosomes contribute to bone remodeling, including osteogenesis, osteoclast genesis and angiogenesis [16]. In this study, miR-424-5 was highly expressed in BMSCs-derived exosomes, and miR-424-5 overexpression inhibited osteoblast differentiation. In addition, we found that WIF1 was a target of miR-424-5p, which inhibited osteogenesis through WIF1/Wnt/β-catenin.

Exosomes, one of the smallest extracellular vesicles, have been shown to carry different nucleic acids, including miRNAs. MiRNAs modulate cell evolvement and metabolism by inhibiting gene expression after transcription [17]. At present, increasing evidences show that exosomes can be used as a new treatment method for tissue repair, which has been extensively studied in cancers [18], Parkinson's disease [19] and liver fibrosis [20]. Similarly, MCS-derived exosomes can mediate the process of bone formation through paracrine, and can be adopted as a new treatment direction of osteoporosis [21], which has been confirmed in ONFH [22] and osteoarthritis [23]. Therefore, it is of great significance to study the role of exosomes in bone differentiation.

Recently, miRNAs in exosomes have been proved to contribute to osteogenic differentiation. Yang S et al. used microarray to analyze the expression profiles of adipogenic stem cell (ADSC) exosomes from undifferentiated and osteoblastic human cells, and found that 201 miRNAs are up-regulated and 33 miRNAs are down-regulated, confirming that expression changes in exosome miRNAs promote osteogenic differentiation of ADSCs [24]. Studies have confirmed that miR-21 was overexpressed in MSC-derived exosomes, and the overexpressed miR-21 dampens osteogenesis by inhibiting SMAD7 [5, 25]. In addition, Chen S et al. discovered that exosomes from human adipose-derived stromal cells (hASCs) overexpressing miR-375 enhance osteogenic differentiation [26]. Nevertheless, Yang JX et al. found that over-expression of miRNAs attenuates osteogenic differentiation. The results showed that osteoclast-derived exosomes containing miR-23a-5p effectively abate osteoblast differentiation by abating Runx2 and elevating YAP1-mediated MT1DP [27]. Thus, miRNAs can either promote or inhibit osteogenic differentiation by binding different downstream targets. As one member of miRNAs, miR-424-5p is one of the core miRNAs for tension-induced bone formation, and is related to the pathology of subchondral bone (SCB) sclerosis [28]. Although the abnormal expression of miR-424-5p in bone diseases has been studied, its role in bone differentiation and specific mechanisms are rarely reported. Therefore, we probed into the miR-424-5p level in BMSCs-derived exosomes and its role in osteogenic differentiation. As a result, miR-424-5p is highly expressed in OP(Exo), and its overexpression inhibits the expression of OPN, OCN and Runx2, ALP activity, and osteogenic differentiation. Nevertheless, the specific mechanism needs to be further clarified in the following experiments.

WIF1 is a recombinant human WNT inhibitor, which directly interacts with various Wnt ligands. The epigenetic promoter methylation of WIF1 results in transcriptional silencing and WNT signaling up-regulation [8]. By comparing the unfused and prematurely fused tissues, it was found that WIF1 is differentially expressed, verifying that WIF1 is related to the differentiation ability of human calvarium tissues *in vivo* [29]. In addition, Cho SW et al. found that dose-dependent treatment with WIF1 attenuates the transcriptional activity of β-catenin/T-cell factor (TCF) in the cell line, and it also dampens the mRNA levels of Runx2, Type I collagen, ALP, and osteocalcin in C3H10T1/2 cells [30]. Thus, the WIF1/Wnt/β-catenin signaling pathway inhibits osteogenic differentiation, which is consistent with our results.

However, the mechanism of WIF1/Wnt/β-catenin signaling pathway interaction with exosomes enriched miR-424-5p remains elusive. Therefore, we studied the regulatory mechanism between miR-424-5p and the WIF1/Wnt/β-catenin signaling pathway. It was discovered that miR-424-5p is overexpressed, while WIF1 is knocked down in BMSCs-derived exosomes. Considering that their biological functions were confirmed, but the interaction between them remains elusive, we conducted a dual-luciferase reporter assay and RIP assay. Interestingly, the results manifested that miR-424-5p binds to WIF1. Moreover, overexpressing WIF1 inhibits the miR-424-5p expression in osteoblast differentiated cells, while BMSCs-derived exosomes partially reverses the osteogenic effect of BMSCs-derived exosomes in osteoporosis patients through WIF1 overexpression.

Here, the manuscript discovered miR-424-5p is overexpressed in BMSCs-derived exosomes, and miR-424-5p overexpression abates osteogenic differentiation, while the overexpression of WIF1 partially reverses the osteogenic inhibition of BMSCs-derived exosomes. Our findings suggested that intervention of miRNA in exosomes may be a new direction for the treatment of osteoporosis.
